# MicroRNAs: Key Regulators in the Central Nervous System and Their Implication in Neurological Diseases

**DOI:** 10.3390/ijms17060842

**Published:** 2016-05-28

**Authors:** Dan-Dan Cao, Lu Li, Wai-Yee Chan

**Affiliations:** Faculty of Medicine, School of Biomedical Sciences, The Chinese University of Hong Kong—Chinese Academy of Sciences Guangzhou Institute of Biomedicine and Health Joint Laboratory on Stem Cell and Regenerative Medicine, The Chinese University of Hong Kong, Shatin, N.T., Hong Kong 999077, SAR, China; caodandan@link.cuhk.edu.hk (D.-D.C.); greenpear0804@hotmail.com (L.L.)

**Keywords:** microRNA, regulation, CNS development, neurogenesis, neurological diseases

## Abstract

MicroRNAs (miRNAs) are a class of small, well-conserved noncoding RNAs that regulate gene expression post-transcriptionally. They have been demonstrated to regulate a lot of biological pathways and cellular functions. Many miRNAs are dynamically regulated during central nervous system (CNS) development and are spatially expressed in adult brain indicating their essential roles in neural development and function. In addition, accumulating evidence strongly suggests that dysfunction of miRNAs contributes to neurological diseases. These observations, together with their gene regulation property, implicated miRNAs to be the key regulators in the complex genetic network of the CNS. In this review, we first focus on the ways through which miRNAs exert the regulatory function and how miRNAs are regulated in the CNS. We then summarize recent findings that highlight the versatile roles of miRNAs in normal CNS physiology and their association with several types of neurological diseases. Subsequently we discuss the limitations of miRNAs research based on current studies as well as the potential therapeutic applications and challenges of miRNAs in neurological disorders. We endeavor to provide an updated description of the regulatory roles of miRNAs in normal CNS functions and pathogenesis of neurological diseases.

## 1. Introduction

### 1.1. Biogenesis of miRNAs

miRNAs are a large family of short single strand RNAs consisting of ~22 nucleotides. Since miRNAs are regulators controlling gene expression post-transcriptionally, biogenesis and turnover of miRNAs are tightly associated with proper expression of genes.

**Figure 1:**
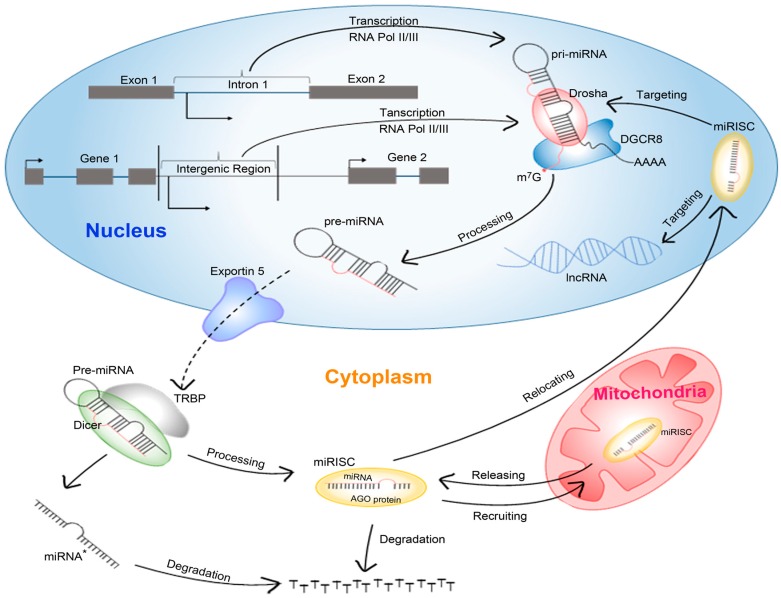
miRNA biogenesis and action. Pri-miRNA is typically transcribed from intron or intergenic region by polymerase II or polymerase III (Pol II or Pol III). In the nucleus the pri-miRNA is recognized and cleaved by Drosha and its partner DGCR8 to generate the ~70 nucleotides of pre-miRNA. The nuclear export of pre-miRNA molecules into the cytoplasm is mediated by Exportin 5 (XPO5) where they are further processed by Dicer with the aid of TAR RNA binding protein (TRBP) to generate the duplex of miRNA:miRNA*. Generally, the miRNA* is released to be degraded, while the miRNA is loaded into Agronaute (AGO) protein to form the miRNA-induced silencing complexes (miRISC) which could regulate the gene expression post-transcriptionally. When the target mRNAs are unavailable, the miRNA would also decay after being released from the miRISC. In some cases, the miRISC is recruited to mitochondria or relocated to nucleus, at which it can target diverse targets including pri-miRNAs and long non-coding RNAs (lncRNAs).

miRNA biogenesis is a multistep process facilitated by several enzymes (Figure 1). Briefly, the primary miRNA (pri-miRNA) is transcribed by RNA polymerase II or polymerase III either from intergenic region or from intronic region of the hosting gene [1,2,3]. Then the pri-miRNA is cleaved in the nucleus by the endoribonuclease Drosha, a member of the RNase III proteins, into ~70 nucleotide stem-loop precursor miRNA (pre-miRNA) [4]. Drosha in complex with DGCR8 (DiGeorge critical region 8) binds to the double-strand stem of pri-miRNA and cleaves the 3′ and 5′ strand of pri-miRNA by its RNase III domain. The product, pre-miRNA, is exported to the cytoplasm with the aids of an exportin5-Ran-GTP complex and further processed by another member of RNase III protein—Dicer into ~22 base pair miRNA/miRNA* (guided strand/passenger strand of the same hairpin structure) duplex [5,6]. The structural features of pre-miRNA, including the 3′- and 5′- open terminus with 2 nucleotides 3′-overhang, and the terminal loop structures favor the accurate recognition and further precise cleavage of Dicer. Additionally, the double-strand stem structure greatly promotes the binding between target pre-miRNAs and Dicer [6,7]. In most cases, the guide strand (miRNA) of the pre-miRNA duplex with less thermodynamics stability will be selectively embedded into AGO (Agronaute) protein to form the miRISC (miRNA-induced silencing complex), in which miRNA can bind to target mRNA via incomplete base-pairing, while the passenger strand (miRNA*) of pre-miRNA will be degraded. However, in a few cases, two strands of a pre-miRNA would co-accumulate as a sister-pair and are employed in targeting two different sets of mRNAs separately [8]. Regardless of asymmetry of thermo-stability, either strand of a miRNA/miRNA* could be loaded into miRISCs depending on the availability of substrates (mRNAs) in specific tissues [9].

### 1.2. Turnover of miRNAs

As described above, the passenger miRNAs will be degraded after strand selection. Moreover, when the target mRNAs are not available or highly complementary to miRNAs, the miRNAs are also detached from miRSICs and hence decay (Figure 1) [10]. Therefore, the steady expression level of miRNAs is regulated by both biogenesis and degradation, although the latter has received limited attention so far, partially due to the long decay period of which can be ten times longer than that of mRNAs [11]. However, in the process of development, the expression of miRNAs changes quickly when cell types convert, which is in accordance with their regulatory roles in cellular development [10]. Remarkably, the varied expressions of only mature miRNAs but not their pri-miRNAs or pre-miRNAs at different stages support the view that miRNAs are modulated to decay appropriately in response to different cellular environments. Interestingly, the degradation of miRNAs in neurons is more variable than in other cell types [12]. The decay rate of several miRNAs, including miR-219 and miR-132, were accelerated by blocking glutamate receptors in neurons, whereas that of miRNA cluster miR-183/96/182 were inhibited under the same treatment condition [12,13]. The quick decaying response of miRNAs to physiological stimuli, including illumination change and synaptic stimulation, implicates that active miRNAs metabolism might strongly support the neuronal functions and plasticity [14].

### 1.3. Mechanisms of miRNA Regulation

**Figure 2:**
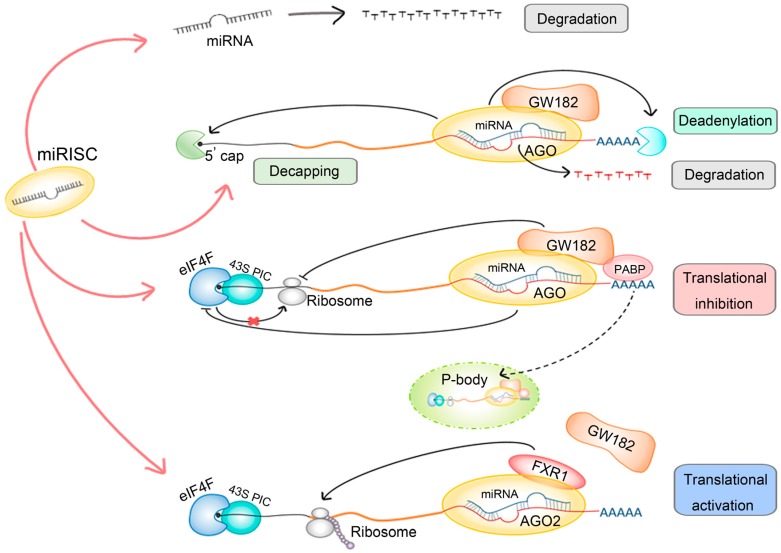
miRNA regulatory mechanisms. When the target mRNAs are not available, or not highly complementary to miRNAs, although rare in mammals, the miRNAs would be degraded. In most of cases, the miRISC, which generally constitute of miRNA, AGO protein and miRNA-induced silencing complexes (GW182), would target the 3′-UTR of mRNA. The imperfect complementary bet-ween miRNA and the 3′-UTR of mRNA would destabilize the 3′ poly(A) tail and 5′ cap of the mRNA and lead to degradation. Besides, the binding of miRISC with auxiliary protein poly(A)-binding protein (PABP) to mRNA could cause the translational inhibition via repressing activity of the eIF4F complex and the 43S pre-initiation complex (PIC). The dynamics structure P-body (processing body) could also sequester the target mRNA from the translation process. In a few cases, the translation of the mRNA in ribosome is promoted instead of inhibited by the miRISC. The red line represents major pathways of miRISC, the dark line represents interaction between different components, and the dotted line represents alternative way of translation inhibition processed by P-body.

The dynamic changes of gene expression during stem cell differentiation have been reported, at least partially, to be regulated by miRNAs [15]. Basically, an individual miRNA can target up to thousands of mRNAs by the sequence complementarity between 5′ end of miRNAs and 3′-UTR (untranslated region) of mRNAs [16]. Generally, miRNAs are believed to inhibit gene expression by either inducing mRNA deadenylation, degradation, or translational repression (Figure 2) [17]. Briefly, after miRNAs guide the miRSICs to their targets, the mRNAs will be cleaved by AGO proteins if the base-paring is perfect. Otherwise, the imperfect sequence matching between miRNAs and mRNAs will cause deadenylation and decapping of mRNAs, leading to a loss of poly(A) tail in the 3′ end and capping structure in the 5′ end [18]. The remaining destabilized mRNAs are further cleaved by either exonucleases or endonucleases [19]. Alternatively, the miRNAs can prevent translational initiation via the interaction between AGO and initiation factors [20]. GW182 (glycine tryptophan protein of 182 kDa) protein, is also an important component and downstream effector of miRSCSs that can interact with PABP (poly(A)-binding protein) to recruit several auxiliary proteins that corporately inhibit translational initiation [21,22,23]. For instance, upon the binding of miRSICs, the assembly of eIF4F complex, which is able to recruit 40S subunit to form a 43S pre-initiation complex, will be blocked to inhibit translation of mRNA [24,25]. Interestingly, there was a debate that whether all isoforms of eIF4A proteins are mediated by miRNAs. As one study proposed, the eIF4A2 could facilitate miRNAs to repress more than initiate translation; whereas another study claimed that both eIF4A1 and eIF4A2 would be dissociated from the target mRNA under the miRNA-mediated repression [26,27]. Except for these canonically repressing ways, miRSICs were also indicated to prevent mRNA translation with the facilitation of P-bodies (processing bodies) that sequester target mRNAs from translation processing and hence destroy them or keep them for further processes [28,29,30]. Non-canonically, without PABP, miRSICs execute translational activation function (Figure 2). In this manner, GW182 misses its interaction with AGO2 and allows another protein Fragile X mental retardation protein 1 (FXR1) to form micro-ribonucleoprotein (microRNP) complex to exert its translational activation function [19,31,32].

### 1.4. MicroRNA Regulation in Canonical and Non-Canonical Ways

As described previously, the conventional function of microRNAs is to repress its target transcripts. The inhibitory function is fulfilled by complementary sequence between the 6–8 nucleotides at the end of miRNA and the 3′-UTR sequence of its target mRNA [16]. For example, the widely studied neuronal microRNA miR-9* targets the 3′-UTR of *BAF53a* to repress *BAF53a* expression [33].

In addition to this extensively studied canonical way of miRNA regulation, several non-canonical regulatory mechanisms have been discovered. First, the binding site of target mRNA for miRNA can also be regions other than the 3′-UTR, including the 5′-UTR [34], the promoter [35] and the coding sequence [36]. For example, the transcriptome-wide miR-155 binding map revealed binding sites outside the 3′-UTR [37]. Bioinformatics tools that take all possible binding regions into consideration have been developed [38,39]. This feature reminds us that we need to be careful not restricting the target region to the 3′-UTR when studying miRNA function. Secondly, miRNAs do not only have inhibitory functions but can also effect translational activation (Figure 2). An example is miR-369-3. miR-369-3 was first discovered to up-regulate translation dependent on the presence of FXR1 and AGO2 by Vasudevan *et al.* [31]. In addition, miR-373 has been reported to induce gene expression by binding to the promoter, thus competing with the promoter-binding repressor [35]. More detailed summary of miRNA-mediated upregulation could be found in another review [19]. The fact that increasing number of publications reveals the role of miRNAs as activators makes it important to study miRNAs function more carefully. Thirdly, miRNAs are not only confined to the cytoplasm (Figure 1). A number of reports have revealed the presence of particular miRNAs in animal cell nuclei [40,41,42,43]. In 2011, Jeffries *et al.* described the localization of a substantial number of human miRNAs in the nucleus of neural stem cells. They found that some miRNAs have higher score in the nucleus than in the cytoplasm [44]. This non-canonical localization of miRNAs suggests these molecules to have novel functions. Fourthly, miRNAs do not only regulate mRNA expression. Although the specific mechanism for the transportation of mature miRNAs into the nucleus remains largely unknown, the discovery of the presence of miRNAs in the nucleus is intriguing and indicative that the nuclear miRNAs can function non-canonically. MicroRNAs have been proposed to regulate miRNA directly by targeting the primary transcripts (Figure 1) [45]. For example, miR-709 has been shown to suppress miR-15a/16-1 maturation by targeting the pri-miR-15a/16-1 [46]. MicroRNAs have also been modeled to regulate long noncoding RNA (lncRNA) [45]. Hansen *et al.* reported that lncRNAs could be functionally targeted by miRNAs with experimental evidence [47]. In summary, so far much effort has been placed on studying the canonical models of miRNA in the past decades. However, more and more evidence have been accumulated for the non-canonical regulatory actions of miRNAs. It is promising and exciting to further study the non-canonical mechanisms of miRNA regulation. We believe and fully anticipate that the investigation of novel aspects of miRNA function will significantly reinforce our understanding of the complex gene regulatory network.

## 2. MicroRNA Regulation and Function in the Central Nervous System

**Figure 3:**
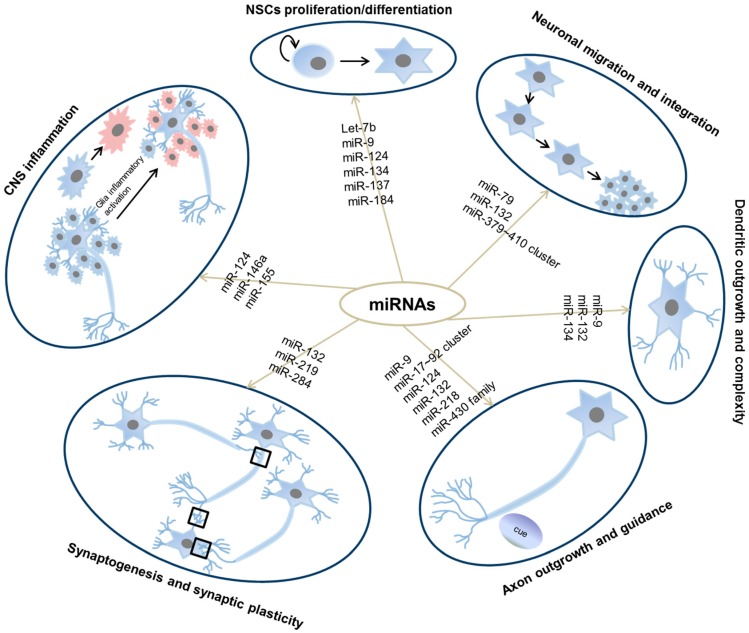
Roles of miRNAs during neuronal development. Listed are miRNAs covered in this review that are functional in each stage of neuronal development. Red indicates activated microglia. Black square indicates synapses.

Development of the mammalian nervous system is the result of a series of coordinated events including transcriptional networks, cell signaling, cell actions and structural organization [48]. The regulatory network produces interconnected glia and neurons. In average there are 86 billion neurons in human brain [49]. They form intricate neural circuitry to communicate with each other through electro-chemical signals. Among the sophisticated multilayered regulatory networks in the nervous system, miRNAs emerge as crucial regulators during embryonic development, adult neurogenesis and upon environmental stimuli. miRNAs have been shown to be abundantly expressed in the brain [50]. Moreover, many miRNAs display dynamic and specific temporal and spatial patterns of expression during brain development and in adult brain [51,52]. In addition to expression evidence, a lot of studies identified versatile functions of specific miRNAs in both invertebrates and vertebrates during neural development and brain activities [53]. In this section, we will review different ways of miRNA regulation and how miRNAs are regulated in the CNS, and summarize the reported functions of miRNAs in the CNS accordingly (Figure 3).

### 2.1. MicroRNA Regulation in the CNS

In the introduction section, we generally presented microRNA regulation in canonical and non-canonical ways all of which are also involved in the CNS. As a result, in this part, we only summarize other aspects of microRNA regulation in the CNS.

#### 2.1.1. Crosstalk between miRNA and Epigenetics

Epigenetic changes including DNA methylation and histone modification have been shown to play important roles during brain development [54,55]. Emerging evidence reveals that epigenetic changes and miRNA regulation are inter-related with each other making the gene regulatory network in the CNS more complex and robust.

DNA methylation and histone modification can regulate miRNA expression directly or indirectly in non-neuronal system. In DNA methyltransferases knockout cell lines, the expression of about 10% of the miRNAs was regulated [56]. Similarly, histone deacetylase (HDAC) inhibitor treatment in a cell line changed the expression of a subset of miRNAs [57]. Treatment by both DNA demethylation agent and HDAC inhibitor caused miRNA expression changes in bladder cancer cells [58]. In turn, miRNAs can also regulate DNA methylation and histone modification. For example, members of DNMTs have been reported to be targeted by miRNAs: miR-29 targeting *DNMT3a/3b* [59], miR-199a-3p targeting *DNMT3a* [60] and miR-148 targeting *DNMT3b* [61] through binding to their 3′-UTR or coding region. Moreover, miR-449a has been demonstrated to directly downregulate *HDAC-1*. This crosstalk between miRNA and epigenetics also exists in the CNS. In 2010, Szulwach *et al.* studied the crosstalk between miR-137, *MeCP2* and *Ezh2* [62]. MeCP2 is a member of the family of DNA methyl-CpG-binding proteins (MBDs) [63]. It is essential for normal function of nerve cells and is involved in several diseases [64]. *De novo* mutation of *MeCP2* causes Rett syndrome [65], while overexpression of *MeCP2* in monkey brain resulted in autism-like behaviors [66]. In mouse neural stem cells (NSCs) with *Mecp2*-deficiency, altered miRNA expression has been identified, among which miR-137 was further studied in detail [62]. miR-137 was shown to be an important modulator in adult neurogenesis and the expression of miR-137 was identified to be epigenetically regulated by MeCP2 through altering the epigenetic status in the chromatin surrounding miR-137 resulting in premature miR-137 expression [62]. Furthermore, miR-137 functionally targeted Ezh2, a histone methyltransferase and member of the PcG protein family [62]. This kind of suppression of *Ezh2* caused an overall reduction in H3K27-trimethylation [62]. Another study published in 2010 provided additional evidence in crosstalk between miRNA and epigenetics [67]. In that study, miR-184 was identified as a microRNA functioning to regulate the balance between proliferation and differentiation of neural stem cells by targeting *Numblike*, a known regulator of brain development. Interestingly, miR-184 is regulated by MBD1, an epigenetic transcriptional repressor. Taking together, the communication between miRNA and epigenetics elicits a more comprehensive regulatory network in the CNS. It will be vital to further study how they interact with each other in normal physiology in the CNS and in pathogenesis in neurological diseases.

#### 2.1.2. MicroRNA Regulation by Mitochondria

Mitochondria are responsible for the production of most of the cell’s energy through respiration. In addition, mitochondria also function in cell signaling, cellular differentiation, and cell survival. There is clear evidence showing that mitochondrial function loss happened to the CNS after brain injury [68]. In addition, several studies suggest the potential link between mitochondrial function and miRNA activity [69,70,71,72].

In a short highlight [73], the authors suggested that mitochondria are prime candidates for regulating miRNA activity based on two pieces of evidence, namely, thermodynamics of miRNA:mRNA interaction and the interaction between mitochondria and other cellular compartments, organelles and cytoplasmic foci is important for energy distribution, signaling and homeostasis. They proposed several ways how mitochondria affect miRNA activity and function. First, mitochondria serve as a warehouse in which miRNAs are stored because a number of miRNAs have been reported to be present in mitochondria. For example, miR-155 has been shown to be enriched in mitochondria in mouse liver [69], while several other miRNAs including miR-142-3p/5p, miR-146a are enriched in human hippocampal mitochondria [72]. Secondly, mitochondria are utilized as vehicles to deliver miRNAs to corresponding compartments in cells to tightly regulate gene expression. This localized and highly regulated mRNA expression or translation is particularly essential in the CNS. Thirdly, the dynamic network of mitochondria-mitochondria-cellular compartments provides an intricate platform for delivery or exchange of miRNAs. This manner ensures precise control in cellular gene expression in specific context or domain.

Considering the important roles of neuronal mRNA transportation, local protein synthesis and mitochondria in many events of the CNS [74,75], it is worthy not only pointing out this highly novel dimension of gene regulation by the interaction and crosstalk between miRNA and mitochondria, but also further studying how these interactions mediate responses to cellular cues in the CNS. This undiscovered mechanism in gene regulation might advance our knowledge in miRNA regulation in the CNS and bring effective means to control abnormal events happened in the CNS in future.

#### 2.1.3. MicroRNA Regulation by Neuronal Activity

Neuronal activity results in synaptic strength alteration by inducing cellular and molecular changes. Many studies have shown that some miRNAs are specifically enriched in dendrites and synaptosomes indicating the activity-dependent regulation of miRNAs in the CNS [76,77,78,79]. Several screening studies have also shown altered miRNA levels (increased or decreased) when neuronal activity is altered by chemical stimulation or electrical stimulation either *in vitro* or *in vivo* [80,81,82,83]. In addition, specific miRNAs have been proved to be regulated by neuronal activity through different pathways. For example, miR-132, the first and widely studied miRNA in activity-dependent regulation, has been identified to be increased after different types of neuronal activity including brain-derived neurotrophic factor (BDNF) and KCl intake in cultured neurons [84], and cocaine intake in particular brain regions of living animals [85]. The induction of miR-132 involves CREB (cAMP response element-binding protein, a crucial stimulus-induced transcription factor), NMDA receptor, CaM kinase and MEK-ERK pathways [86,87]. Moreover, other miRNAs such as miR-212, miR-134 and miR-124 have also been identified to be regulated by neuronal activity via different molecular pathways, thus to a degree ensures precise local responses to various types of neuronal activity [88].

In regard to the biogenesis of miRNAs, together with the outcomes of miRNA changes (increase or decrease) after neuronal activity, possible mechanisms for regulation of miRNAs have been proposed [89,90]: (i) Localization of precursor miRNAs is regulated by neuronal activity. Bicker *et al.* found that the dendritic localization of precursor-microRNA-134 upon BDNF treatment was mediated by the DEAH-box helicase DHX36 through interaction with the pre-miR-134 terminal loop [91]. In addition to dendritic localization, the selectively localization of specific pre-miRNAs to axons of sensory neurons upon injury have also been discovered [92]. However, the molecular mechanisms of pre-miRNA localization are still required more extensive study; (ii) Transcription of primary miRNAs is regulated by neuronal activity. For example, in miR-132 gene regulation, CREB is activated to bind to CRE responsive elements in the promoter of pri-miR-132 and induce miR-132 transcription [93,94]; (iii) Biogenesis of miRNAs is regulated by neuronal activity. It has been demonstrated that in non-neuronal systems, the microprocessor complex is stimulus-regulated which subsequently affect miRNA generation [95]. However, this initial evidence requires further studying in neuronal system; (iv) Turnover of miRNAs is regulated by neuronal activity. A miRNA turnover study in neurons showed that regulated turnover happened without any changes in Drosha or Dicer protein levels which suggests that enzymes critical for miRNA degradation and stability should be involved [12]; (v) The repressive function of miRNAs within miRISC is regulated by neuronal activity. The reversibility of miRNA-mediated repression at the level of miRISC is particularly appropriate for the regulation of local protein synthesis upon neuronal activation. However, further research investigations in this aspect are still required; (vi) Sequestration of miRNAs is regulated by neuronal activity. Recently, studies have shown that circular RNAs could work as sponges for miRNAs leading to release of the function of sponged miRNAs [96,97]. For example, a circRNA derived from Sry transcript could sponge miR-138 in testes [97]. Since circRNAs have also been found in specific brain regions, it may be possible that miRNA could be regulated by neuronal activity through circular RNA regulation. However, it still needs to be proved.

The synaptic plasticity and activity are crucial for neuronal function and higher order of cognitive function [98]. Thus, it is a prerequisite to decipher the mechanisms for miRNA regulation by neuronal activity. This will lead us to a better understanding the role of miRNAs in the diversity and complexity of brain function, and benefit us in the development of therapeutic strategies in neurological diseases utilizing miRNA intervention.

### 2.2. MicroRNA Function in the CNS

During mammalian development, NSCs derived from neuroepithelial cells and radial glia cells lining the neural tube give rise to the entire CNS [99]. The discovery of NSCs in adult brain showed that adult neurogenesis also exists [100,101]. Adult NSCs are somehow different from embryonic NSCs in their neurogenic potential. In this section, we will summarize miRNA function in different stages of neuronal development (Figure 3, Table 1).

#### 2.2.1. MicroRNA Function in Embryonic Neural Development

The role of miRNAs in neural development was first discovered by knockout experiments of essential enzymes for miRNA biogenesis. Zebrafish embryos with dicer mutants showed severely affected brain development including impaired formation of neurocoel and neural tube, reduced formation of brain ventricles and so on [122]. In mice, conditional Dicer deletion at late embryonic stages affected neuron migration in the cortex and precursor differentiation in the spinal cord [130]. More conditional knockout mutants were used to confirm additional roles of miRNAs in brain development [131,132]. Most importantly, in addition to these enzyme knockout studies that merely reflect general role of miRNAs in neural development, the functional role of individual miRNA was also studied. For example, overexpression of miR-430 family in Zebrafish could rescue some of the defects in brain development caused by dicer depletion [122].

#### 2.2.2. MicroRNA Function in NSCs Proliferation and Differentiation

While microRNAs have been generally demonstrated to play essential roles during neural development by interfering with the miRNA biogenic machinery, overexpression or inhibition studies of individual miRNAs have delineated the roles of a number of miRNAs in regulating proliferation and differentiation of neural stem cells (NSCs). For example, miR-134 and miR-184 have been demonstrated to regulate neural progenitor maintenance and proliferation, while let-7 family miRNAs (let-7a/let-7b), miR-9, miR-137 and miR-124 have been implicated in promoting neuronal differentiation (reviewed by Bian *et al.*) [102]. Among them, miR-9 and miR-124, the most abundant miRNAs in the brain, have been extensively studied and their complex regulatory network has been well summarized in a detailed review [103]. One miRNA can target hundreds of mRNAs. As a result, to further study the downstream target is very critical in understanding the regulatory mechanisms of miRNAs in defined function. Here, we take miR-124 as an example to show in what manners one miRNA can regulate NSCs proliferation and differentiation.

As shown in 2005, administration of miR-124 in Hela cells caused gene expression shift which resembled that of brain [133]. Later, several independent studies investigated the regulatory mechanisms of miR-124 in different systems. In 2006, Conaco *et al.* studied the upstream regulator of miR-124 [134]. They found that RE1 silencing transcription factor (REST), a transcription repressor that inhibits neuronal gene expression in non-neuronal cells, could repress miR-124 expression in murine cell lines. In 2007, Visvanathan *et al.* demonstrated that miR-124 promoted neuronal differentiation by suppressing SCP1 (small C-terminal domain phosphatase 1) in chick developing spinal cord [104]. SCP1 is an anti-neural factor that functions together with REST to form a REST/SCP1 complex. The study suggested a negative feedback loop between REST/SCP1 and miR-124. In the same year, Makeyev *et al.* identified that miR-124 induced nervous system (NS) specific alternative splicing by targeting the splicing regulators *PTBP1* and *PTBP2* in murine cell line and in embryonic mice [105]. miR-124 represses *PTBP1* expression which subsequently releases the inhibitory roles of PTBP1 on PTBP2. PTBP2 then promotes NS-specific alternative splicing pattern. miR-124 was also found to ensure neuronal differentiation by repressing two endogenous targets *LAMC1* and *ITGB1* in chick embryos [106]. Another study in 2009 showed that miR-124 could target *Dlx2*, *Jag1* and *Sox9* in the subventricular zone (SVZ) in adult brain to regulate neurogenesis [107]. Most recently, miR-124 has been revealed to regulate neuronal differentiation of mesenchymal stem cells by targeting *Sp1* (Specificity protein) [108].

From the above discussion, we know that miR-124 can function in different system to ensure neuronal differentiation. The mechanisms are very complex. Those downstream targets may form complex regulatory loops resulting in different phenotypic effects or function in different systems. Thus, more efforts are required to uncover and study more targets for each miRNA.

#### 2.2.3. MicroRNA Function in Neuronal Migration and Integration

Neuronal migration and integration are fundamental processes that are critical for the architecture of the brain. These two processes are intimately linked. In the newly emerging area of induced pluripotent stem cell (iPSCs) transplantation for CNS-related diseases, neuronal migration and integration are undoubtedly crucial for the newborn neuron to find the right position to integrate into the existing neuronal circuitry.

A few examples have shown that miRNAs play roles in neuronal migration and integration. In *C. elegans*, Pedersen *et al.* reported that mir-79 (an ortholog of mammalian miR-9) pathway was required to regulate proteoglycan biosynthesis to direct neuronal migration [109]. In mice, Rago *et al.* identified the miR379-410 cluster as regulatory miRNAs of *N-cadherin* which is known to be regulating neuronal migration in the developing neocortex [111]. Another example of miRNA in neuronal integration is miR-132. The knockdown of miR-132 impaired the functional neuronal integration in the adult Dentate Gyrus [110]. It was further shown that this mediating process might be through inflammatory regulation since the genes increased by knockdown of miR-132 were enriched in inflammatory and immune function [110]. Detailed study about the miR-132 regulatory mechanism in neuronal integration is required.

#### 2.2.4. MicroRNA Function in Dendritic Complexity

Dendrites are one of the two types of protrusions from the soma of a neuron. Each neuron has one axon and many dendrites. Typically, axons carry signals away from the cell body toward the post-synaptic cell while dendrites receive signals from the pre-synaptic cell. The ability of dendrites to convey information depends on the architecture of dendritic tree. In the past decades, emerging evidence has shown the critical regulatory function of miRNAs in dendritic development and maturation.

In 2006, Schratt *et al.* identified the expression of miR-134 in dendrites in rat hippocampal neurons [76]. They also revealed that miR-134 negatively regulated dendritic spine size in hippocampal neurons via inhibiting translation of *Limk1* mRNA. Another group performed a comprehensive study about the role of miR-132/212 in adult hippocampus in mice [113]. They found that miR-132 is the predominant microRNA of the cluster miR-132/212 the knockout of which resulted in decreased dendritic length and arborization. Later, the role of miR-132 in reducing dendritic complexity and spine density was reproduced by Pathania *et al.* in olfactory bulb neurons [114]. Jasinska *et al.* demonstrated that the regulation of dendritic spine structure by miR-132 might be fulfilled via targeting *MMP-9* (matrix metalloproteinase-9) [115]. A further detailed regulatory network of miR-132 in regulating hippocampal dendritic spine formation was revealed by Dhar *et al.* [116]. They found that Leptin could increase spine formation in mouse hippocampus through LepR signaling which is required for normal hippocampal spine formation. miR-132 was found to be regulated by CREB which is activated by Leptin. miR-132 regulated dendritic growth via inhibiting *P250GAP* translation. They found that CREB-mediated miR-132 transcription was critical for Leptin function in dendritic spine formation. Additionally, miR-9 has also been discovered to play a role in dendritic development [112]. Reduced dendritic length and complexity were observed upon miR-9 loss both *in vitro* and *in vivo*. This kind of dendritic growth defects might be mediated by the downstream target *REST* of miR-9. However, considering the complex regulatory network involving miRNAs and mRNAs, further studies are required to decipher the mechanisms.

#### 2.2.5. MicroRNA Function in Axon Outgrowth and Guidance

Axon outgrowth is very important for neuronal circuitry assembly. An axon is firstly specified and extended by one of the neurites formed around the soma of the neuron. Upon the guidance cues from the environment, axons are navigated to reach their synaptic targets. Both the axon outgrowth and guidance are identified to be regulated by miRNAs.

Axon outgrowth could be regulated at the neurite formation stage. miR-132 can inhibit p250 GTPase-activating protein to induce neurite sprouting in cortical neurons [93]. Axon outgrowth can also be affected by local protein synthesis. miR-9 was found to locally repress *Map1b* in axon which finally reduced axon length [117]. On the other hand, miR-17~92 cluster was identified to downregulate *PTEN* and activate mTOR pathway which promotes axon outgrowth [119].

Axon guidance includes key steps as long-range guidance, fasciculation and targeting. Collectively, axon guidance is modulated by guidance molecules such as Ephrins, Semaphorins, Slits, Netrins, morphogens, growth factors and cell-adhesion molecules [135,136]. Although the role of miRNAs in regulating axon guidance is rarely studied, emerging evidence shows us that miRNAs are still valuable molecules in the complex regulatory network. The first report of miRNAs functioning in long-range axon navigation was performed by Pinter and Hindges [121]. In the study, they conditionally abolished miRNAs function by generating Dicer mutant mice resulting in aberrant guided retinal ganglion cells (RGCs) axons. miR-218 which is expressed in axons may further explain this phenomenon because it can target *Robo1/2* (a receptor involved in the process of RGCs axon guidance) in other system. In addition, scientists found that miR-9 mutant mice showed misrouted thalamocortical and corticofugal axons in telencephalic development [118]. Although the mechanism of how miR-9 causes the abnormal phenotype remains unclear, they proposed that miR-9 may regulate the patterning of guidepost cells. Usually, axons start their pathfinding journey with pioneer axons. The dysregulation of miRNAs can affect the formation of fasciculation. In 2005, Giraldez *et al.* reported Dicer zebrafish mutants exhibited defasciculated axons which could result in aberrant axonal trajectory [122]. Interestingly, exogenous miR-430 family members could partially rescue this phenotype [122]. In the previous example, defasciculation in RGCs was also observed [121]. The modulatory mechanisms of miRNAs still require further study. The last step of axon guidance is targeting which is definitely essential. In this part, miRNAs have also been shown to play important roles. Knockdown of miR-124 in RGCs led to inappropriate stalling axons within the optic tectum because of wrong axonal response to Sema3A expression at the right place [120]. These axons wrongly targeted regions in the ventral border. On the molecular level, miR-124 indirectly up-regulated *Neruropilin-1* expression via mediating its downstream target *coREST*.

In the axonal area, miRNAs emerge as important regulatory molecules. However, the exact roles played by miRNAs still remain largely unknown. More efforts are needed to elucidate miRNA function in axonal outgrowth especially in axonal guidance.

#### 2.2.6. MicroRNA Function in Synaptogenesis and Synaptic Plasticity

Synaptogenesis is the formation of synapses between neurons as the axons and dendrites grow. Major events during synaptogenesis include axon guidance, dendritic growth and assembly of large numbers of synaptic protein complex. The role of miRNAs in axon guidance and dendritic growth has been discussed in the previous section. Considering the major regulatory mechanism of miRNAs is targeting mRNAs to mediate their expression or translation, it is reasonable to propose that miRNAs could function in regulating the assembly of synaptic protein complex. This notion is supported by the identification of miRNAs in the synaptodendritic compartment [78,79]. In addition, in *D. melanogaster*, mutations in *Dicer* and *Armitage* caused aberrant synaptic synthesis of crucial regulatory proteins, such as CAMKIIa [137].

Synaptic plasticity is the ability of synapses to change in synaptic strength. It can result from specific patterns of synaptic activity, as well as the alteration of the number of neurotransmitter receptors in synapses. This biological process is thought to contribute to learning and memory and higher order processing [138]. MicroRNAs have been shown to play important roles in synaptic plasticity, as well as higher cognitive function [89,139]. At fly neuromuscular junction, miR-284 was shown to control the abundance of glutamate receptor subunits namely GluRA and GluRB implicating the role of miR-284 in determination of postsynaptic strength [124]. Interestingly, the orthologous subunit *GluR1* and *GluR2* have been identified to be locally translated in mammalian hippocampal neurons [140]. However, further efforts are needed to discover involvement of miRNAs in this regulation. In a higher order, miRNAs could be correlated to complex behavior. In mice, the inhibition of miR-132 and miR-219 in the suprachiasmatic nucleus of the hypothalamus led to disturbed circadian rhythm, probably through dysregulation of clock genes [86]. miR-132 was also correlated to learning and memory processes. Inhibition of miR-132 *in vivo* led to impairment in the storage of temporally associated information [123].

Synapses are increasingly believed to be essential players in the aetiology of a number of neurological diseases. Considering miRNAs participate in almost every stage in synaptic development, the studying of miRNAs in these diseases is promising. The information is summarized in the last part of this review.

#### 2.2.7. MicroRNA Function in CNS Inflammation

CNS inflammation plays a critical role in acute CNS injury and chronic CNS diseases. In the CNS inflammatory system, microglia have been identified as resident innate immune cells which are very important in immune surveillance [141]. The role of miRNAs in CNS inflammation is firstly supported by assaying expression of glia-enriched miRNA, such as miR-223, miR-146a, miR-125b-5p and so on [142,143]. Interestingly, some of them were expressed in a heterogeneous manner in microglia from different brain regions suggesting the key regulatory roles of miRNAs in the behavior of microglia. Moreover, altered expression of miRNAs has been identified in microglia cells after exposure to a pro-inflammatory signal (LPS) [144]. Further mechanism studies identified specific role of specific miRNA in microglia pro-inflammatory function. For example, miR-155, which is regulated by p53, targeted *c-Maf* to promote microglia pro-inflammatory function [129]. Another example is miR-146a, which is induced by presenilin 2 (PS2), can suppress NF-κB transcriptional activity in a negative feedback mechanism [126,127,128]. Dysfunction of PS2 causes decrease in miR-146a that results in the increase of NF-κB transcriptional activity and increases pro-inflammatory cytokine expression. MicroRNA regulating CNS inflammation is also involved in neurological diseases. In 2011, Ponomarev *et al.* found that miR-124, via the C/EBP-α-PU.1 pathway, could inhibit autoimmune encephalomyelitis (EAE) and reduce CNS inflammation through promoting microglia quiescence and deactivating macrophages [125]. These findings suggest the profound effect of miRNA on CNS inflammation and related neurological diseases.

## 3. Implications of miRNAs in Neurological Diseases

MicroRNAs have been shown to contribute to every stage during neural development implicating the possible roles of miRNAs in the pathology of neurological disorders. Indeed, accumulating evidence show that dysregulation of miRNAs happened in different neurological disorders. Generally, neurological disorders can be categorized into three major types: neurodevelopmental disorders, neuropsychiatric disorders, and neurodegenerative disorders. In the following, we will summarize the evidence for roles of miRNAs in neurological disorders by presenting one or two specific disease for each category (Table 2).

### 3.1. Neurodevelopmental Diseases

#### 3.1.1. Fragile X Syndrome

Fragile X syndrome (FXS) is a common inherited form of mental retardation resulted from the defective expression of *FMRP* gene [170]. The mutations in *FMRP* lead to changes in neural morphology and synaptic plasticity [171,172]. Interestingly, several studies indicated that FMRP executes its function through downstream miRNAs at least partially. In Drosophila, it was showed that phenotypes caused by overexpression of miR-124a could be partially rescued by inactivation of dFMR1 (Drosophila homology of FMRP) [145]. In mouse, Edbauer *et al.* identified that FMRP was required for miR-125b and miR-132 effects on spine morphology changes [146]. Moreover, they also showed that FMRP regulated the expression of the NMDA receptor subunit NR2A through miR-125b. Taken together, miRNAs might be important mediators in the FMRP regulatory pathway in FXS.

#### 3.1.2. Rett Syndrome

Rett syndrome (RTT) is an X-linked neurodevelopmental disorder with occurrence primarily in females. Genetically, mutations in *MeCP2* are the main cause of RTT [65]. The implication of miRNA in RTT pathology came from the study performed in 2007 by Klein *et al.* [84]. They used rat as a model and found that loss of *MeCP2* delayed neuronal maturation and synaptogenesis. *MeCP2* was identified as a target of miR-132 and its expression is suppressed by miR-132. Together with the evidence provided by the same group that BDNF induces the expression of miR-132 [93], and that increasing of MeCP2 increases BDNF level, a feedback regulatory loop might be formed to control the *MeCP2* through miR-132. More importantly, a transgenic mouse model with miR-132 overexpression in forebrain neurons manifested significant increase of dendritic spine density in hippocampal neurons, behavior deficits and decreased *Mecp2* level [147]. Conversely, MeCP2 can also regulate miRNAs as its downstream targets. For example, miR-184 was found to be up-regulated by releasing MeCP2 from its promoter binding site in cultured cortical neurons [148]. However, opposite effect (downregulation of miR-184) was identified in the *Mecp2*-deficient mouse brain [148]. The phenomenon may be due to that one miR-184 might be under different regulated manner in different systems. The role of miRNAs in *Mecp2* regulatory pathway is further supported by the fact that altered miRNA expression profile was identified in *Mecp2*-null mice [173,174]. Among these changed miRNAs, a miRNA cluster (miR-379-410) has been implicated in dendritic morphology regulation [149]. Overall, miRNAs have been implicated in *Mecp2*-dependent regulatory network, thus may contribute to RTT phenotypes.

#### 3.1.3. Autism Spectrum Disorders

Autism spectrum disorders (ASDs) are a group of complex neurodevelopmental disorders with heterogeneous phenotypes as well as genetic heterogeneity. ASDs are mainly characterized as having difficulties with social interaction, repetitive behaviors and impairments in communication. FXS, RTT and ASD patients usually have some common features suggesting that miRNAs involved in FMRP-dependent or *Mecp2*-dependent regulatory pathway also contribute to ASD pathogenesis [175]. Indeed, *Mecp2* duplication in transgenic monkeys demonstrated autism-like behaviors [66]. In addition to the indirect inference, direct evidence is provided by a number of miRNA expression profiling studies in ASD patients. Both lymphoblastoid cell line samples and postmortem brain samples were identified with altered miRNA expression comparing ASD patients to normal controls [176,177,178,179]. Furthermore, genetic studies also add up to the possibility of miRNAs regulatory roles in ASD. Microduplication 22q11.2 was more frequently observed in ASD cases than that in controls [180,181]. Interestingly, in this region is located one of the miRNA biogenesis related gene *DGCR8*, as well as one specific microRNA miR-185 which might be likely altered by the microduplication [150]. In a similar way, miR-211 has been found to locate within a genomic region at 15q13.2-q13.3 which has been associated with ASDs [151]. All of the above possible cases in other way remind us that further systematic investigation and specific study of specific miRNA in pathology of ASDs will be definitely necessary.

### 3.2. Neuropsychiatric Diseases

#### 3.2.1. Major Depression Disorder

Major depression disorder (MDD) is one of the most prevalent psychiatric disorders that severely affect the quality of life. Although the pathogenesis of MDD remains largely unknown, a large number of studies of MDD subjects point to the association between neural plasticity and MDD [182,183]. As miRNAs have been shown to be critical in neuronal plasticity, it is reasonable to associate miRNA function with MDD. Actually, several lines of evidence have clearly proved this notion. miRNA profiling in postmortem brains of MDD revealed altered miRNA expression [184]. Further analysis showed that *VEGFA*, a gene implicated in depression is the downstream target of several dysregulated miRNAs [184]. Gene variants studies associated miRNA processing with MDD. He *et al.* identified that polymorphisms in *DGCR8*, *AGO1* and *GEMIN4* were significantly different in their frequency between patients with depression and controls without psychiatric disorders [185]. As we know stress is a critical factor in the development of MDD, studies on stressor-induced effects on miRNAs have been performed which provide additional indirect evidence for the involvement of miRNAs in MDD. Meerson *et al.* identified differentially altered miRNAs in central amygdala and the Cornu Ammonis area1 in rat during acute and chronic stress [186]. Uchida *et al.* studied the effects of early-life stress (maternal separation here) [187]. They found that *REST4* (a splicing variant of *REST*) was increased upon maternal separation. It was suggestive that REST4 might play a role in the development of behavioral vulnerability. Regarding that, overexpression of *REST4* in the medial prefrontal cortex of neonatal mice caused depression-like behaviors in adult mice under repeated restraint stress. The role of miRNAs in depression is reflective by other studies which have identified REST is a factor which regulates the expression of miRNAs, such as miR-124 and miR-9 [134,152]. In another study, Bai *et al.* provided evidence that development of depression behavior could result from maternal separation in rats, and decreased BDNF level was also observed upon this kind of early life stress [153]. Interestingly, expression of BDNF was negatively correlated with miR-16 indicating the regulatory role of miR-16 in the development of depression [153]. Collectively, knowledge of the role of miRNAs in MDD is still in its infancy, more work should be on the way to unravel the puzzle.

#### 3.2.2. Schizophrenia

Schizophrenia (SCZ) is one of the most common psychiatric disorders. Genetics contribute to the etiology of SCZ with family and monozygotic twin study. Structurally, synapses are increasingly recognized as one of the important cellular compartments contributing to the aetiology. Considering the knowledge that miRNAs have been shown to play roles in virtually all steps during synapse development, miRNAs might have potential causative function in SCZs. Expression profiling studies of miRNAs in postmortem brain of SCZ patients have identified many dysregulated miRNAs [188,189,190,191,192]. In addition to these general profiling studies in heterogeneous SCZ patients, detailed miRNA studies were performed on a mouse model Df(16)A+/− carrying a syntenic microdeletion conserved to 1.5Mb human 22q11.2 microdeletion [155]. Human 22q11.2 microdeletion is a well-known genetic risk factor for SCZ [193]. Df(16)+/− mice exhibited morphological changes in dendritic trees and spine density, as well as behavioral and cognitive dysfunctions making it a promising model to study miRNA dysregulation in a disease context [155]. Profiling study identified miRNA changes and 10%–20% of them were downregulated by DGCR8, a gene located in the microdeletion region [155]. More specifically, miR-185 is also included in that region. As displayed in ASDs, 22q11.2 microduplication (not microdeletion) is associated with disease pathogenesis suggesting different miRNA regulatory network in different neurological disorders. Furthermore, a number of genetic studies have provided additional supportive data for the contribution of miRNA in SCZ pathology. For example, a number of genetic variants have been identified affecting genes such as *DICER* and *CYFIP1* [194,195]. As well, genetic variants have also been identified to contain certain miRNAs such as miR-211, miR-484, or to be associated with certain microRNA such as miR-137 [156,157,158]. However, the effect of variants on these indicative factors should be further studied to get final conclusions. In addition, a larger scale miRNAs profiling study should be performed to produce convergent picture for SCZs.

### 3.3. Neurodegenerative Diseases

#### 3.3.1. Alzheimer’s Disease

Alzheimer’s disease (AD) is the most common neurodegenerative disorder, characterized by cognitive dysfunction and neuronal degeneration. The neuropathology includes amyloid plaques and neurofibrillary tangles. To date, four genes including amyloid precursor protein (APP), presenilin 1 (PS1), presenilin 2 (PS2), and apolipoprotein E (ApoE) have been considered to be associated with familial AD [196]. The potential of miRNA regulation in AD attribute to not only altered miRNA expression identified in samples of AD patients [197,198,199], but also a number of miRNAs shown to be involved in regulation of diseases gene products. For example, miR-98 was identified to suppress *IGF1*, a gene involved in APP processing [159]. The study showed that Aβ production was reinforced by overexpression of miR-98 which down-regulated *IGF1*. Another example is miR-124, which was found to inhibit *PTBP1*, a gene regulating the production of APP with skipping exon 7 and 8 in normal neurons [160]. However, decreased miR-124 and increased APP containing exon 7 and 8 were found in AD brains suggesting the role of miR-124 in AD pathology. Other studies revealed miR-26b and miR-34a regulating Tau while miR-146a regulated by Presenilin [126,161,162]. More recently, a study highlighted 6 pathogenic miRNAs (namely miR-7, miR-9, miR-34a, miR-125b and miR-146a) involving NF-κB regulated signaling pathway in AD suggesting the broad regulatory ways of miRNAs in AD [163]. However, regarding the complexity of miRNA-modulated regulatory network, more efforts are required to uncover the stage of miRNAs in AD.

#### 3.3.2. Parkinson’s Disease

Parkinson’s disease (PD) is the second most common neurodegenerative disorder characterized with progressive deterioration of motor function, which is due to progressive loss of dopaminergic neurons. So far, *SNCA*, *LRRK2*, *PARK2*, *DJ1* and *PINK1* are the widely accepted genes for inherited PD. A number of miRNAs have been reported to be altered in PD and were predicted to regulate the associated genes. Among those miRNAs, miR-133b which is enriched in the midbrain was identified to be downregulated in the midbrain of PD patients [164]. Mechanistic analysis showed that miR-133b could target *Pitx3*, an important transcription factor in dopaminergic neuron development [164]. Moreover, let-7 and miR-184* have been found to be involved in the LRRK2 mediated pathogenic pathway [165]. Inhibition of let-7 and miR-184* led to loss of DA neurons and reduce locomotion activity [165]. In another study, it was shown that the induction of miR-205 prevented the neurite growth defects in neurons with *LRRK2* mutation [166]. These results suggest the protective role of let-7, miR-184 and miR-205 for PD. However, further evidence is required to confirm this speculation. The gene, *SNCA*, the most important causative factor of PD, was predicted to be targeted by several miRNAs, such as miR-7, miR-153 and could be regulated indirectly by miR-433 [167,169,200]. A detailed list of miRNAs predictively regulating genes in PD can be found in another review [168]. More work is needed to discover new miRNAs and to fully understand the regulatory roles of the miRNAs in order to depict a more intricate picture of the relationship between miRNAs and PD.

## 4. Therapeutic Applications and Challenges

The previous paragraphs summarized the involvement of miRNAs in different kinds of neurological disorders (NDs) with different levels of evidence. The expression of miRNAs is dysregulated in patient brain compared to normal brain. The genetic evidence has showed that mutations that disrupt miRNA transcription were found in NDs. Specific function of specific miRNA has been discovered to be associated with NDs. Moreover, the manipulation of miRNAs expression could change the phenotypes in some NDs using animal model. Thus, it is reasonable to expect to use miRNAs as therapeutic agents. In fact, miRNAs have already been successfully used in cancer therapy [201]. The small size of miRNAs makes it easier to synthesize and manipulated. In addition, each miRNA can regulate numerous targets of the same pathway thus place it as more effective mediator when compared to manipulating a single gene. Besides the promising future of miRNA as targets to treat NDs, miRNAs could also be utilized as biomarkers. Previous studies showed that brain miRNA could circulate through the body in the exosome without degradation [202,203]. Furthermore, miRNA profiling studies have shown that circulating miRNAs could be used as potential biomarkers for neurological diseases [204,205,206]. Thus, by monitoring miRNAs in the periphery e.g., plasma, serum, blood cells or cerebrospinal fluid, one could potentially predict disease occurrence as well as evaluate prognostic effects. For example, Lopez *et al.* identified miR-1202 to be useful as a predictive marker for drug response in MDD patients [154]. Without accessing to the brain sample which is refractory to access, detection of biomarker in the circulating system is relatively easy and effective in early diagnosis and personalized effective remedy.

Although utilizing miRNAs as therapeutic targets and biomarkers for NDs are very promising, there still exist a lot of challenges. As summarized in this review, only minor downstream targets have been identified for one miRNA, thus implying the ‘off-targets’ effect may cause severe unanticipated responses. In addition, a lot of evidence has showed that one miRNA can function in different stages and in different regions in the brain with different mechanisms making it hard to adopt precise targeting policy and precise delivery. Besides using miRNA as a target, the upstream regulator of miRNAs could also be the object of potential manipulation. However, which one is more effective needs to be further evaluated. Further, due to blood brain barrier (BBB), it may be hard to achieve precise delivery of miRNA to its intended target. Finally, the synthetic technology and deliver technology are also factors that should be taken into consideration. We believe with the rapid advances in systematic drug delivery, successful miRNA-based therapeutics in NDs will be achieved in the near future.

## 5. Conclusions and Outlook

From what we have shown, miRNAs in the past decades have been clearly identified as crucial factors during neural development. However, there still exist major gaps in our knowledge of miRNAs in the CNS to be filled. Firstly, it is now clear that one miRNA can play different roles in different stages of neuronal development or in different regions of the brain. We are far from knowing how a single miRNA is regulated in neurons. From a different angle, this fact also cautions us to be careful in translating the function of a single miRNA. Secondly, several miRNAs can act synergistically. As a result, identifying accurate interacting network is critical for precise understanding of their function. Thirdly, in some aspects of neural development, the role of miRNAs has only started to emerge. For example, in axon guidance, neuron migration and integration, relatively little is known about miRNA function. Fourthly, almost all the mechanistic studies are based on the model organism, animal studies, or *in vitro* studies which are very different from human. We should be very careful to in collecting and deciphering information from these studies for further interpretation and application. Human induce pluripotent stem cells (hiPSCs) may offer an effective model for studying the function and mechanistic roles of miRNAs inhuman [207,208,209]. Efforts in using primate model should also be performed. We believe, with advances in technology, more knowledge about miRNA regulation in the CNS and subsequent clinical usage can be achieved.

With the accumulating evidence implicating the roles of miRNAs in different kinds of neurological disorders, it is compelling to develop miRNA-based therapeutics to treat those complex neurological disorders. However, conveying precise delivery and preventing off-target effects is still challenging. On the other hand, finding early diagnostic and non-invasive prognostic miRNAs as biomarkers for the complex NDs will keep researchers busy for the following decades.

## Figures and Tables

**Table 1:** Relevant miRNAs and their functions in the CNS physiology in this review.

miRNA	Species	Targets/Pathway	Function	References
**NSC Proliferation/Differentiation**				
let-7b	Mouse	*TLX, Cyclin D1*	NSC proliferation and neuronal differentiation	[102]
miR-9	Mouse	*TLX*	NSC expansion and differentiation	[102,103]
miR-134	Mouse	*Dcx, Chrdl-1*	cortical NPC proliferation, neuron migration	[102]
miR-137	Mouse	*LSD1*	NSC expansion and differentiation	[102]
miR-124	Chick	*SCP1*	neuronal differentiation	[104]
	Mouse	*PTBP1*	neuronal differentiation	[105]
	Chick	*LAMC1, ITGB1*	neuronal differentiation	[106]
	Mouse	*SOX9*	neuronal differentiation	[107]
	Mouse	*DLX2, JAG1*	–	[107]
	Human	*SP1*	neuronal differentiation	[108]
miR-184	Mouse	*Numbl*	adult NSC proliferation and differentiation	[67]
**Neuronal Migration and Integration**				
miR-79	*C. elegans*	*SVQ-5, SVQ-7*	neuronal migration	[109]
miR-132	Mouse	–	newborn neuron integration, dnedritic spine density	[110]
miR-379~410 cluster	Mouse	*N-cadherin*	NSC differentiation and neuronal migration	[111]
**Dendritic Outgrowth and Complexity**				
miR-9	Mouse	*REST*	dendritic development, total dendritic length and complexity	[112]
miR-132	Mouse	–	dendrite length, arborization, and spine density	[113]
	Mouse	–	dendritic complexity and spine density	[114]
	Mouse	*MMP-9*	dendritic spine structure	[115]
	Mouse	*p250GAP*	activity-dependent dendritic growth	[116]
miR-134	Rat	*Limk1*	the size of dendritic spines	[76]
**Axon Outgrowth and Guidance**				
miR-9	Mouse	*Map1b*	axonal extension and branching	[117]
	Mouse	–	axon guidance	[118]
miR-17~92 cluster	Rat	*PTEN*	axon outgrowth	[119]
miR-124	*Xenopus laevis*	*coREST*	axon targeting	[120]
miR-132	Rat	*p250GAP*	neurite outgrowth	[93]
miR-218	–	*Robo1/2*	axon guidance	[121]
miR-430 family	Zebrafish	–	axon fasciculation	[122]
**Synaptogenesis and Synaptic Plasticity**				
miR-132	Mouse	–	the storage of temporally associated information	[123]
	Mouse	–	light-induced clock resetting	[86]
miR-219	Mouse	–	circadian period length	[86]
miR-284	Drosophila	*GluRIIA* and *GluRIIB*	postsynaptic strength	[124]
**CNS Inflammation**				
miR-124	Mouse	CEBPα-PU.1	microglia quiescence	[125]
miR-146a	Mouse & Human	NF-κB	pro-inflammatory cytokine expression	[126,127,128]
miR-155	Mouse	*cMAF*	microglia pro-inflammatory function	[129]

**Table 2:** Relevant miRNAs implicated in neurological diseases in this review.

Neurological Diseases	miRNA	Evidence	References
Fragile X Syndrome (FXS)	miR-124a	Involved in *FMRP* gene regulatory pathway	[145]
	miR-125b, miR-132		[146]
Rett Syndrome (RTT)	miR-132	Involved in *MeCP2* gene regulatory pathway	[84,93,147]
	miR-184		[148]
	miR-379-410		[149]
Autism spectrum disorder (ASD)	miR-185	Located in ASD associated microduplication 22q11.2	[150]
	miR-211	Located in ASD associated variant 15q13.2-q13.3	[151]
Major depression disorder (MDD)	miR-9	Involved in *REST* gene regulatory pathway	[152]
	miR-124		[134]
	miR-16	Involved in *BDNF* regulatory pathway	[153]
	miR-1202	biomarker of antidepressant response of MDD	[154]
Schizophrenia (SCZ)	miR-185	Located in SCZ associated microdeletion 22q11.2	[155]
	miR-211	Encompassing SCZ associated genetic variants	[156]
	miR-484		[157]
	miR-137		[158]
Alzheimer’s disease (AD)	miR-98	Involved in APP processing by targeting *IGF1*	[159]
	miR-124	Regulating production of APP isoforms by targeting *PTBP1*	[160]
	miR-26b	Regulating Tau protein	[161]
	miR-34a		[162]
	miR-146a	Regulated by Presenilin	[126]
	miR-7, miR-9, miR-34a, miR-125b, miR-146a	Involved in NF-κB regulated signaling pathway	[163]
Parkinson disease (PD)	miR-133b	Regulating dopaminergic neurons by targeting *Pitx3*	[164]
	let-7, miR-184*	Involved in *LRRK2* regulatory pathway	[165]
	miR-205		[166]
	miR-7, miR-153	Predicted to target *SNCA*	[167]
	miR-433	Regulating *SNCA* indirectly	[168,169]
